# The efficacy of group counselling on perceived stress among infertile women undergoing in vitro fertilization treatment: An RCT

**DOI:** 10.18502/ijrm.v17i1.3821

**Published:** 2019-03-07

**Authors:** Zeinab Hamzehgardeshi, Fereshteh Yazdani, Forouzan Elyasi, Mahmood Moosazadeh, Sepideh Peyvandi, Keshvar Samadaee Gelehkolaee, Maryam Shahidi

**Affiliations:** ^1^Sexual and Reproductive Health Research Center, Mazandaran University of Medical Sciences, Sari, Iran.; ^2^Department of Reproductive Health and Midwifery, School of Nursing and Midwifery, Mazandaran University of Medical Sciences, Sari, Iran.; ^3^Traditional and Complementary Medicine Research Center, Addiction Institute, Mazandaran University of Medical Sciences, Sari, Iran.; ^4^Midwifery Counseling, Mazandaran University of Medical Sciences, Sari, Iran.; ^5^Student Research Committee, School of Nursing and Midwifery, Shahid Beheshti University of Medical Sciences, Tehran, Iran.; ^6^Department of Psychiatry, Psychiatry and Behavioral Sciences Research Center, Addiction Institute, School of Medicine, Mazandaran University of Medical Sciences, Sari, Iran.; ^7^Health Science Research Center, Addiction Institute, Mazandaran University of Medical Sciences, Sari, Iran.; ^8^IVF Ward, Mazandaran University of Medical Sciences, Sari, Iran.; ^9^Department of Reproductive Health and Midwifery, Tehran Nursing and Midwifery Faculty, Tehran University of Medical Sciences, Tehran, Iran.; ^10^Department of Medical Physics, Mazandaran Medical University, Mazandaran, Iran.; ^11^Hazrat-e Maryam Fertility Center (HMFC), Sari, Iran.

**Keywords:** *Infertility*, * Group counselling*, * Perceived infertility stress.*

## Abstract

**Background:**

One of the stressful and critical experiences that threat the individual, family, marital, and social stability is infertility.

**Objective:**

To identify the effects of midwifery-led counselling programs on the perceived stress of the women undergoing assisted reproductive treatment.

**Materials and Methods:**

In this randomized clinical trial, 50 infertile women who underwent in vitro fertilization treatment for the first time were enrolled in two groups. The intervention group received six sessions of group counselling by M.Sc. midwifery of counseling student and the control group received only the routine care. All participants filled Newton's standard questionnaire before and at the time of puncture, embryo transfer and the pregnancy test.

**Results:**

The mean ± SD scores for the perceived infertility stress before the intervention in the control and the intervention groups were 167.92 ± 12.14 and 166.75 ± 13.27, respectively. The mean of perceived stress after intervention at the time of oocyte puncture in the control and case group were 177.12 ± 19.37 and 115.75 ± 13.88, at the time of embryo transfer were 179.40 ± 18.34 and 118.08 ± 15.37, and at the time of pregnancy test was 183.76 ± 14.97 and 120.50 ± 16.24, respectively. The perceived stress of infertility after intervention were statistically significant in the two group (p ≤ 0.001).

**Conclusion:**

Group counselling is one of the effective methods for reducing the perceived stress in the women undergoing assisted reproductive treatment.

## 1. Introduction

Infertility is identified as a stressful and critical experience (1, 2) that affects all aspects of an individual's life (2). The global infertility rate is between 12 and 15% (3), and the total mean of infertility in Iran has been reported as 13.2% (4). Perceived infertility stress is defined as signs that are caused by infertility, analogous to most symptoms of post-traumatic stress disorder (5). Infertility-related stress includes the interaction of the physical conditions and medical interventions, which may last for years and relapse subsequent to any diagnostic or therapeutic interventions (6).

Hence, along with the development of infertility treatment technologies, dealing with the psychological pressure of infertile women is extremely important for the treatment procedure and contributes to the improvement of the treatment outcomes (7). Up to now, various methods have been developed to prepare the patients and improve their mental status, including psychotherapy, relaxation, stress management, cognitive-behavioral intervention, grief management, as well as sexual and marital disorder treatment (8). An example is a study into the effects of cooperative infertility counselling on infertile women's perceived stress (1); another example is the development of stress management support groups for couples undergoing in vitro fertilization (IVF) treatment (9). However, no Comprehensive studies have been conducted based on a definite treatment protocol with a focus on reducing the stress of infertile women undergoing IVF treatment.

In an era where financial concerns become increasingly important, individual therapy is substituted by group therapy (10). The benefit of Group therapy counselling is: sharing experiences, receiving information, improving communication skills, learning relaxation techniques, or providing other forms of mental support (11). Furthermore, group counselling reduces social isolation of infertile women (12); on the other hand, according to the guidelines, a person who takes care of an infertile women has to be totally aware of the etiology of infertility as well as treatment procedure and interaction culture of the region so that they can help these women to cope with the disease effectively (13, 14).

Since psychological counselling and perceived social support for infertile women have considerable effects on individuals' physical and mental status (15), and based on the international guidelines that states that fertility treatment centers are required to provide infertile with psychological counselling (16), and in addition due to the lack of exhaustive studies into the effects of midwife-led supportive counselling programs on women's perceived stress, the present study was conducted to identify the effects of midwifery-led counselling programs on the perceived stress of the women undergoing assisted reproductive technology treatment. The results of the study could improve the infertile women's physical/mental health, enhance treatment outcomes and, as a consequence, increase the total fertility rate.

Therefore, the present study aimed to investigate the effect of group counselling on perceived stress among infertile women who underwent the IVF treatment.

## 2. Materials and Methods

### Participants

`In this randomized clinical trial, women who underwent IVF/ICSI due to primary infertility and were referred to public and private infertility center in Sari, North of Iran were enrolled. The inclusion criteria were women aged < 45 yr with primary infertility, undergoing IVF or ICSI, holding Iranian nationality, literate, having no intention to use donated egg/embryo or surrogacy, not being an adoptive parent, not consulting a psychiatrist/psychologist since one month prior to the study, not suffering any major psychological disorder at the time of the study (diagnosed by a psychologist) and not taking any psychiatric medications, not having had any severe family disputes since one week prior to the study, not suffering any systemic diseases such as diabetics, hypertension, hyperlipidemia, and thyroid diseases (as obtained from the medical history, anamnesis, and their self-report(1). All women with IVF cycle cancellation by the physician, severe family dispute, death of a beloved one or experiencing any other tragedies during the study, positive pregnancy test during the study, the necessity to take psychotropic medications during the study, and two weeks of absence or more from counselling sessions (1, 17) were excluded.

According to the table of random numbers, our participants were divided into two groups: the Intervention group (*n* = 25) and the control group (*n* = 25).

To avoid bias, sampling was done in each center individually for intervention and control group. When the sampling of the intervention group was carried out at one center, at the same time, the control group was sampled at another center, and after completing half of the samples, two centers were replaced for the intervention and control samples.

### Intervention 

This study is a part of a larger study. The counselling sessions were conducted and monitored by an M.Sc. student of midwifery counseling as well as the research team. The group counselling sessions were held on a weekly basis for six weeks. Each session lasted 2 hours and were divided into two sections; the first section lasted half an hour and was based on the group's process section (18), and the second section lasted one hour and a half, which included psychological counselling and education based on the psychoeducation model (19).

The first session included the required information on the study and its aims, the genito-reproductive system, definition of infertility and its etiology, and the impact of stress and negative emotions on hormonal and physiological changes of the reproductive system. At the end of the first session, relaxation techniques were taught, and its effects and usefulness on the improvement of treatment outcome were elaborated. A combined relaxation technique was adopted using Jacobson's and Benson's relaxation techniques (20). In the first session, body relaxation techniques were taught.

The focus of the second session was on improving the management of emotions such as grief, shock, feelings of guilt, contempt, embarrassment, and hopelessness. In this session, counselling was concerned with identifying negative self-conscious thoughts, recognizing cognitive errors, and improving the negative sense. However, the head and neck relaxation were taught. Then, educational pamphlets, relaxation audio CDs, and the participant's relaxation practice report form were handed out to the participants; then, they were asked to practice the relaxation techniques once a day and at least five times a week and fill in the forms (1). The research team reminded and conducted the follow-up exercises by sending SMS messages.

In the third session, communication skills and the process of interacting with others were discussed. This counselling session included a deeper understanding of communication, increasing knowledge and awareness about family communication challenges, examining healthy and unhealthy communication patterns and increasing knowledge about communication skills and solving a positive issue. The fourth session was concerned with coping skills, including developing the concept of healthy and unhealthy coping skills, enhancing awareness of individual coping skills, gaining knowledge about the importance of healthy coping skills, and facilitating group learning and peer support (21).

The fifth session was related to stress management and adjustment. In this session, understanding stress and its importance, finding stress warning signs, improving the understanding of individual physical responses to stress, discovering healthy stress management practices, facilitating group learning, and supporting healthy lifestyles (nutrition and exercise) were carried out, and the sixth session was allocated to other aspects of treatment and using third-party reproduction (religious, ethical, and legal issues) (21).

At the end of each session, the subjects were asked to practice relaxation before the consultant midwife. After the end of counseling sessions and the embryo transfer, the women were asked to practice relaxation techniques at home as per the instructions received, on a daily basis and at least five days a week, until the pregnancy test (two weeks after embryo transfer).

The women in the control group were only provided with the routine counseling, which included explanations about the fertility treatment method and how to use infertility drugs and their complications, but they were asked to inform the research team if they needed a psychologist or psychiatrist. Due to ethical concerns, a pamphlet was handed out to the subjects in the control group commenting on different assisted reproductive technologies.

### Outcomes

To measure perceived infertility stress and the main outcomes of the study, Newton's Infertility Stress Questionnaire was completed for both groups, on the first day and before the first session as a pre-test. Then, it was completed at the day of oocyte puncture, the embryo transfer, and the pregnancy test as a post-test.

### Measuring tools

One of the data collection tools in this study was demographic questionnaire, including personal and family information on age, education, spouse's education, occupation, spouse's occupation, and marriage duration. Questions on infertility information and its treatment included the awareness about duration of their infertility, duration of treatment, cause of infertility, and the treatment expectancy.

Another data collection tool was Newton's infertility stress questionnaire. Designed in 1995 by Newton, it is a 46-item scale with 5 subscales, including social concerns (10 items), sexual concerns (8 items), relationship concerns (10 items), rejection of child-free lifestyle (8 items), and need for parenthood (10 items). The answers were given on a Likert scale from 1 to 6. The overall score of the questionnaire was between 46 and 276. The higher score demonstrate that the stress is higher.

Newton used Cronbach's Alpha coefficients to ensure the reliability of the questionnaire and reported the reliability of each subscale as follows: social concerns 87%, sexual concerns 77%, relationship concerns 82%, rejection of child-free lifestyle 80%, need for parenthood 84%, and overall stress 93%. Additionally, the validity index of the instrument in terms of replicability was 0.83%. The validity of the instrument was assessed using the correlation among the five scales with a mean of 0.45.

As a result, Newton's infertility stress questionnaire is a reliable self-report with good convergent and divergent validity. Therefore, this instrument can be used to diagnose infertility stress (22). This questionnaire was administered to a sample of 30 infertile individuals (15 men and 15 women) in Iran by Alizade and colleagues to examine its reliability; the Cronbach's Alpha for social concerns was turned out to be 0.78, sexual concerns 0.77, relationship concerns 0.78, rejection of child-free lifestyle 0.75, need for parenthood 0.84, and overall stress 0.91 (5).

The Newton's infertility stress questionnaire in Alizade's study (5) was used in the present study due to its convenient reliability. The permission for the use of tools was obtained on June 25, 2015 from Farahani (the corresponding author). Although in this study, the authors used participants' present form of counseling sessions and their relaxation practice report form.

### Ethical consideration

The study protocol was approved by the Ethics Committee of Mazandaran University of Medical Sciences, Sari, Iran (IR.MAZUMS.REC.95.2316). A written informed consent was obtained from all participants.

### Statistical analysis

The final sample size was determined according to the results of the author's pilot study. The mean and the standard deviation for the number of oocytes in the experimental and control groups after pilot study were equal to 13.70 ± 5.20 and 8.80 ± 2.48, respectively. The sample size with a 99% confidence level, 90% test power, and two-sided test using the G-power software was used to estimate 50 women (randomized equally to two groups).

The SPSS software (Statistical Package for the Social Sciences, Version 21.0, SPSS Inc, Chicago, Illinois, USA) were used to analyze the data. To examine the distribution of the data, Kolmogorov–Smirnov test was used. To assess the homogeneity of the two groups with respect to the demographic and confounding variables, Chi-squared test, and independent* t*-test were used. To compare the stress mean score of the two groups, independent *t*-test was used. p-value ≤ 0.05 was considered as the significant level.

## 3. Results

**Figure 1:**
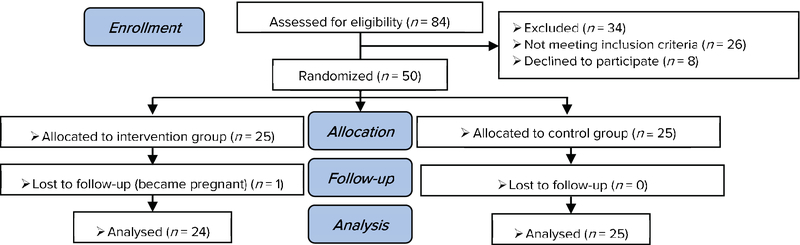
Consort flow diagram of study.

A total number of 50 women with primary infertility participated in this study in two groups. One women in the intervention group was excluded from the study because of pregnancy. Finally, 49 women were analyzed in two groups: intervention group (*n* = 24) and control group (*n* = 25) (Figure 1). There were no significant differences in age, education level, spouse's education level, occupation status, spouse's occupation status, cause of infertility, marriage duration, the length of time one knows about their infertility, and the duration of treatment between two groups (Table I).

**Figure 2:**
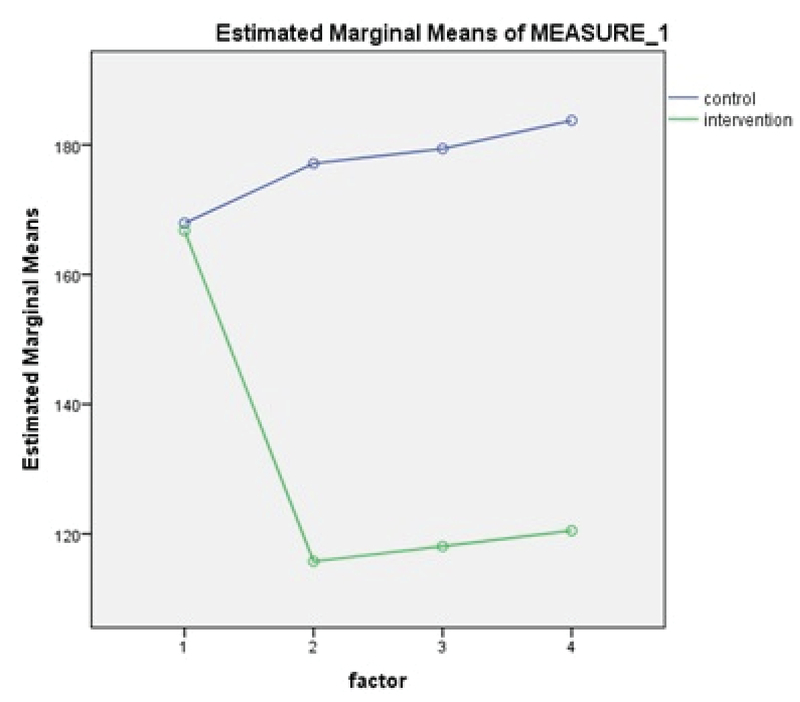
Comparison between the mean and standard deviation of Newton's infertility stress in the intervention and control groups, before and after the intervention in three situations – day of puncture, day of transfer, and day of the pregnancy test.

The main results show that the perceived infertility-related stress had no significant differences between the two groups before intervention (p = 0.749). Whereas, after the intervention, the perceived infertility-related stress rate has shown a statistically significant reduction (p < 0.001). On the contrary, in the control group, not only the perceived infertility-related stress had no decrease, but also its mean score increased in the forthcoming situations (Table II, Figure 2).

**Table 1:** Characteristics of the study population.


**Variable**		**Intervention group**	**Control group**	**p-value**
Age*				
	20–29	9 (37.5)	11 (44)	0.609
	30–39	14 (58.3)	13 (52)	
	40–45	1 (4.2)	1 (4)	
Level of education*			
	Reading and writing ability	5 (20)	2 (8)	0.439
	High school diploma	9 (38.3)	11 (44)	
	College education	10 (41.7)	12 (48)	
Spouse's education level*			
	Reading and writing ability	2 (8.4)	3 (12)	0.751
	High school diploma	7 (29.2)	9 (36)	
	College education	15 (62.4)	13 (52)	
Occupation status*			
	Housekeeper	19 (79.2)	17 (68)	0.427
	Working	5 (20.8)	8 (32)	
Spouse's occupation status*			
	Employee	15 (62.5)	12 (48)	0.371
	Free	6 (25)	11 (44)	
	Farmer	3 (12.5)	2 (4)	
Cause of infertility*			
	Male factor	10 (41.7)	11 (44)	0.263
	Female factor	5 (20.8)	2 (8)	
	Both	6 (25)	4 (16)	
	Unknown	3 (12.5)	8 (32)	
Marriage duration**		5.02 ± 1.86	5.98 ± 1.83	0.076
Length of time one knows about their infertility**		3.52 ± 1.85	4.04 ± 1.64	0.304
Duration of the treatment**		2.47 ± 1.31	3.06 ± 1.66	0.183
Note: *Data presented as frequency (%); **Data presented as mean ± SD; Independent student's *t*-test and chi-square.				

**Table 2:** Comparing the mean of perceived stress of infertility in separate groups in infertile women.


**Variable**	**Intervention group**	**Control group**	**p-value**
Perceived stress of infertility	Meanwhile	166.75 ± 13.27	167.92 ± 12.14	0.749
	After the intervention, day of puncture	115.75 ± 13.88	177.12 ± 19.37	0.000
	After the intervention, day of embryo transfer	118.08 ± 15.37	179.40 ± 18.34	0.000
	After the intervention, pregnancy test day	120.50 ± 16.24	183.76 ± 14.97	0.000
Note: Data presented as mean ± SD; Independent Student's t-test.				

## 4. Discussion

According to the results of the present study, there was a significant difference of the perceived infertility-related stress score between the two groups (p < 0.001). Latifnejad and colleagues (collaborative counselling on the perceived infertility-related stress in women undergoing IVF) (1), Cassill (brief group treatment in couples) (9), and Chan and co-workers (psychosocial group intervention in infertile woman was effective in decreasing infertile women's stress on the ovarian stimulation and embryo transfer days) (23) were in line with this study in reducing stress. In one study, Kheirkhah and colleagues indicated the effect of group counseling on increasing women's adjustment to infertility compared to the control group (17).

Furthermore, focusing on the effects of cognitive counseling and relaxation on reducing the anxiety of women undergoing GIFT and ZIFT, Gharaie and co-workers demonstrated a significant reduction in the anxiety level of infertile women after counseling (24). Gürhan and colleagues examined the effects of nursing counselling on depression in infertile women. They showed that counselling had no significant effects (25). Although depression is different from perceived stress, it is worth noting that Gürhan's study included three counselling sessions held in 12-day intervals, and it concluded that the length and nature of the intervention have considerable effects on improving infertile women's mental status.

Sexton and colleagues showed that the intervention based on the internet can reduce the infertile women's perceived stress, but this reduction did not show any statistically significant difference between the intervention and control (26). The Internet-based nature of the study could account for the underlying reason of the insignificance of the statistics as far as there were many confounding factors in these interventions that were beyond the limits of researcher's ability for homogenization. On the contrary, Cousineau conducted an Internet-based study and reported a significant reduction in the sub-scale of social concerns in Newton's infertility stress questionnaire for the intervention group; however, the overall infertility-related stress score showed no significant difference (27).

Schmidt and colleagues developed an educational program to improve interactions and reduce the couples' stress. They show that there was no significant difference in stress reduction (28), which might be explained by the fact that stress reduction cannot only rely on education; therefore, other aspects such as counseling and psychological support should be considered.

Faramarzi and co-workers compared the effects of cognitive behavioral therapy with the control group who were treated with Fluoxetine. They showed that both methods reduced stress in infertile women, where the reduction was more significant in the counselling group compared to the control group (2), which is in line with the results of the present study.

In one study, Lukse and others indicated the effects of group counseling on grief and that after intervention, the frequency of feeling grief reduced in the intervention group (29). Boivin in one review showed psychological interventions in infertility (group counselling with focus on teaching and learning skills (e.g., relaxation)) had a significantly positive effect on the range of psychological intervention results relating to issues such as stress reduction, expression of feelings, and discussions on infertility-related emotions (30). Frederiksen showed in a meta-analysis study that, like cognitive behavioral therapies, mental interventions for couples seeking fertility treatment could be useful in reducing mental stress and improving fertility treatment (31). This has been documented in Boivin's meta-analysis study, which showed that stress could lead to a failure in infertility treatments (32).

Other results from this study demonstrated that after the intervention, the level of perceived infertility stress in the intervention group decreased, and on the day of transplantation and the day of pregnancy testing, although slightly increased, it was still lower than before the intervention. Meanwhile, the control group not only did not show this decrease but also an increase at later times. Therefore, stress shows an upward trend on embryonic transfer days and in pregnancy testing. One of the studies consistent with the findings of this study is the study of Lancastle and colleagues showing that stress was highest on the days before the pregnancy test (33). This can be because women are constantly faced with mental conflicts before they undergo a pregnancy test and before they are pregnant or not. As a result of this uncertainty about the outcome of the treatment, women tend to become more worried and distressed.

However, a number of studies such as those conducted by Latifnejad and colleagues (2) and Sexton and colleagues (26) showed that the stress in the control group was less than that at the beginning of the study. These studies have concluded that on the days of following the transfer, women consider themselves fertilized and, as a result, it reduces their stress. Therefore, further studies are needed in this regard.

Since from the beginning of the present study, the subjects were monitored and supported by the researcher, based on a systematic protocol, and due to the complementary stress reduction techniques like relaxation, the reduction in perceived infertility-related stress is justifiable.

It is recommended to perform interventions with a larger sample size and with the presence of couples to achieve more generalizable results in the future studies. The limitations of this study included that infertility perceived stress being self-report. In addition to the facts that self-report of infertility stress can be lower-estimate due to the social desirability bias. We did not use biomarkers to detect infertility stress.

Furthermore, considering the results of the study indicating a significant increase in the women's stress during embryo transfer and pregnancy test, it is suggested to carry out interventions to decrease the stress and consequently improve the infertile women's quality of life. Finally, owing to the importance of the issue and the large number of studies conducted into it, it is recommended that fertility treatment centers provide patients with counseling so that they engage in psychological therapies in addition to the physical ones when in need.

## 5. Conclusion

Group counseling is one of the effective methods for reducing the perceived stress in women referred to fertility treatment centers. The results of the study considerably contribute to the research and clinical practice and can be introduced to the officials in the infertility treatment center through reducing stress and improving outcomes of the treatment of infertile women.

### Limitation 

It is believed that people who participated in the study have a better mental status than those who refused to participate. The researcher tried to consent the sample by explaining the consultation process. Although low inclination to participate in group counseling sessions in infertile women has been one of the limitations of the study, during the sampling, the researcher explained the summary of the sessions to the sample and tried to satisfy them to participate in the study.

##  Conflict of Interest

The authors of this article have no conflicts of interest.
